# Notes and News

**Published:** 1890-10-18

**Authors:** 


### Notes and News.

Collected and Communicated.

Mr. E. Roberts, M.R.C.S., on retiring from the position of House Surgeon to the Manchester Royal Eye Hospital, has been presented by the nurses with a handsome timepiece, and by the servants with a brass inkstand. Mr. Roberts has been appointed Honorary Assistant-Surgeon to the hospital.

We are all rather inclined to be proud of a row of carriages waiting at our door: even the consulting physician is not above looking out occasionally between the curtains to note the number of cockades on the hats of the waiting coachmen. The feeling is shared by the shopkeeper: you can see him stand rubbing his hands inside the glass door, and gazing with a satisfied smile at the long line of carriages which has brought him customers. Then there is the line of cabs outside a theatre or restaurant at night, and if a novelist wants material for a realistic novel, he might do worse than join the ranks of these cabdrivers, and listen to their talk during the weary hours of waiting. There are also the long lines of market carts coming to Covent Garden in the cold grey twilight of early dawn, but the very strangest string of vehicles anyone ever beheld gathers outside the out-patient department of the Children's Hospital in Great Ormond Street. No cockades here, and no coachmen; neither are there any horses, though once or twice we have seen a donkey. No, it is a long string of perambulators and handcarts of a most extraordinary description. Not the rubber-tyred, be-hooded baby carriages of the West-end, but good old-fashioned perambulators, shaped like a bath-chair, and with thick wooden wheels. And there are mail-carts, but of home manufacture, and not bearing much resemblance to those we see in Kensington Gardens, handbarrows, wheelbarrows, trollies used for taking home the washing, and sometimes a coster's barrow. Such is the row of vehicles which can be seen outside a children's hospital on out-patient day, and the busy man, who has no time to go inside, can learn the lesson of the work being done by the hospital by a mere glance at the quaint group of carriages awaiting the return of the wee patients from under the doctor's hands.

At the annual meeting of the Northampton General Infirmary, the Rev. Canon Hull presented the following report: "The number of patients during the year has been—In-patients, 1,870; out-patients, 9,202. Of the former, 631 were cured, 409 relieved, and 140 remained in the house on August 1st. The total number of in-patients has been 123 more than in the previous year; the daily average, 145; and the average period of residence has been 5 weeks and 6½ days. The expenditure has amounted to £7,948 19s. 3d., which is £1,230 14s. 11d. more than that of the preceding year. This increase is chiefly accounted for by the fact that the sum expended under the head of repairs amounts to more than £900 than in last year." There was some discussion as to the giving of letters in return for collections, but the question dropped.

The inquiry into hospital abuse at Birmingham was resumed on October 8th. Mr. Jordan Lloyd, visiting surgeon to the Workhouse Infirmary, and honorary surgeon to the Queen's Hospital and the Children's Hospital, said that urgent cases were always admitted to the Workhouse Infirmary irrespective of position. Apart from serious emergencies, admission was secured by relieving officers, who inquired into the circumstances of the applicants. All persons at entering saw the workhouse doctor, who drafted sick persons to the infirmary, and others to the workhouse, and by law all were then paupers. In-patients and out-patients treated were invited to pay for attendance if they could afford it. He believed the system there was absolutely free from abuse. At the Children's Hospital all urgent cases were admitted, and a registration fee of 6d. guaranteed fourteen days' attendance. The number of out-patients was increasing. His chief complaint was that so many of the cases were too trivial for hospital treatment, many being the result of dirty habits and parental neglect. Only about one case in five required expert treatment. Parents who could afford to pay for medical attendance often sent their children there. Sometimes well-to-do tradespeople sent them in the charge of poor women who representsd them to be their own. He mentioned a case of this sort, the father being a publican in a good position. Somewhat similar cases were discovered at the Queen's Hospital.

The Bradford Children's Hospital was opened by Mr. S. C. Lister on October 8th; there was a large gathering, but rain unfortunately marred the proceedings. Dr. Gilchrist Burnie (the hon. sec.) gave a short history of the movement of which the new hospital is the outcome. He said that the movement originated seven years ago in the taking over by a public committee of a small private hospital that had been formed in their own home by the All Saints' Sisters of Margaret Street, London. This happened in the year 1883, and the hospital was started in some upper rooms in the three houses in Hanover Square occupied by the sisters, who for four years retained the management of it under the committee. It soon became apparent that not only did the hospital meet a great want in the town, but that the public were interested in a movement that had for its object the benefit of the most helpless class. This interest culminated in a great bazaar, which resulted in the receiving of about £5,000 for the funds of the institution. This sum enabled the hospital to be at once removed to its late premises in Springfield Place, and at the same time justified the Board of Management in looking about for a site on which to erect a permanent building worthy of the cause. This, after much labour and search, they at last found in the present one, and at once set about erecting the building, whose corner-stone was laid in May, 1889, by the gentleman who had just opened the completed building, and who on that occasion notified his interest by contributing the munificent sum of £5,000 to the funds. This £5,000, with £2,000 contributed by the Fever Hospital trustees, had enabled the Board of Management to perfect the original scheme, and to announce that the new building, which they believed to be the most complete and perfectly designed hospital in England, both as regarded construction and fitting, to be free from debt.

The following vacancies are announced this week, the amount of the salary in each case being given after the name of the institution:—

**Resident Medical Officers.**

Evelina Hospital for Sick Children, House Surgeon—£70, board, etc.
North-West London Hospital, Assistant Medical Officer—board, etc.
Great Northern Central Hospital, House Physician—£60, board, etc.
West London Hospital, House Physician and House Surgeon—board, etc.
Brompton Consumption Hospital, Assistant Medical Officer—£50, board, etc.
Lancashire County Asylum, Assistant Medical Officer—£105, rooms, etc.
Flintshire Dispensary, House Surgeon—£120, rooms, etc.
Birmingham City Asylum, Assistant Medical Officer—£120, board, etc.
Birmingham City Asylum, Clinical Assistant—board, etc.
Nottingham Boro' Asylum, Clinical Assistant—board, etc.
Liverpool Dispensaries, Head Surgeon—£200, rooms, etc.
Roxburgh District Asylum, Assistant Medical Officer—£100, board, etc.
Whitechapel Infirmary, Assistant Medical Officer — £150, rooms, etc.

**Non-Resident Medical Officers.**

Norfolk and Norwich Hospital—Surgeon and Assistant Surgeon.
Nottingham General Hospital, Honorary Physician.
Parish of Kilmonwaig, Medical Officer—£50.
Parish of Portree, Medical Officer—£71.
Taunton and Somerset Hospital, Honorary Physician.

The Austrian Sanitary Board has pronounced against a scheme for the establishment of drunkards' homes for curative purposes. The probability of rescuing habitual drunkards was in their opinion so small that it did not warrant special legislation and heavy expenditure.

A report of the conference on "Alcohol and Childhood," held recently under the auspices of the Church of England Temperance Society (the Duke of Westminster in the chair), which excited so much attention by reason of the number of influential medical men attending it who had not hitherto identified themselves with the temperance movement, is about to be published by the society in a separate form.

At the Hampstead Vestry Hall on Saturday, Mrs. Garrett Anderson, M.D., presided at a meeting at which Miss R. Goodman delivered an address on "The Importance of Physical Culture." Mrs. Anderson spoke of the extreme value of physical exercises, and disagreed with Miss Goodman, who had remarked that English girls could not walk well. She thought they were at the top of the tree in that respect.

We have received from Dr. Conry a copy of his letter to the Local Government Board. We have not room to publish it entire, but gladly give the following extract, which explains why Dr. Conry refused to resign: "Your Board is well aware, and I know from costly experience, that a series of accusations have from time to time been brought against me, and which, like the last one, on investigation have fallen to the ground. On the other hand, the whole course of my administration has frequently been the subject of commendation in official reports to your Board. However irritating these accusations may have been to you, they have never in any one instance been on my initiation, and I altogether decline to be made a victim to other people's obtrusiveness on your official time and attention. If the decision at which you have arrived were to be accepted unchallenged it would be an intimation to all public authorities acting under your jurisdiction that charges, however malicious and unfounded, have only to be sent to your Board sufficiently often to induce it, from a sense of weariness, to dismiss any official who may, and through no fault of his own, be personally obnoxious to the complaining authority. Having given the above explanation, I cannot accede to the request you have made, and must respectfully leave your Executive the responsibility to complete an act of cruel injustice."

